# Supplementary material for: The acquisition of rmpADC can increase virulence of classical Klebsiella pneumoniae in the absence of other hypervirulence-associated genes
Source: bioRxiv. 2025 Sep 21:2025.09.20.677538. Preprint. [Version 1] doi: 10.1101/2025.09.20.677538 (PMC12458134; doi:10.1101/2025.09.20.677538)
Supplement: 1 [file NIHPP2025.09.20.677538V1-supplement-1.pdf]

bioRxiv preprint doi: https://doi.org/10.1101/2025.09.20.677538; this version posted September 21, 2025. The copyright holder for this preprint (which was not certified by peer review) is the author/funder, who has granted bioRxiv a license to display the preprint in perpetuity. It is made available under aCC-BY 4.0 International license.

Table S1. Primers used in this study.

Primer #	Sequence 5 - 3	Use	Reference
SMS015	CATCGCCTTCTATCGCCTTCTTG	pEW103 flank	This work
SMS016	GGATTATTTAGGGAAGAGTGAC	pEW103 flank	This work
SMS031	TTGGCCTGCAAGGCCTCCTTTGTTGAACAATTCCATG	amplify *rmp3*	This work
SMS034	GTTTTGCTGCCCTCGAGGGACACCAAAAGTTATACCATC	amplify *rmp3*	This work
SMS046	TTGGCCTGCAAGGCCTCTTTGTTGAACAATTCCATGC	amplify *rmp1*	This work
SMS047	GTTTTGCTGCCCTCGAGGGAAACAAAAAGCTATACCATC	amplify *rmp1*	This work
KW370	AGTTAACTGGACTACCTCTGTTTC	*rmpA*-Fwd qRT-PCR	This work
KW371	TCCTGCAGTCAACCAATACTC	*rmpA*-Rev qRT-PCR	This work
gyrB-Fwd	CCGAGCTGAACGAGAAAGAA	*gyrB*-Fwd qRT-PCR	This work
gyrB-Rev	GTGGGTACGTACGCGAATAA	*gyrB*-Rev qRT-PCR	This work
